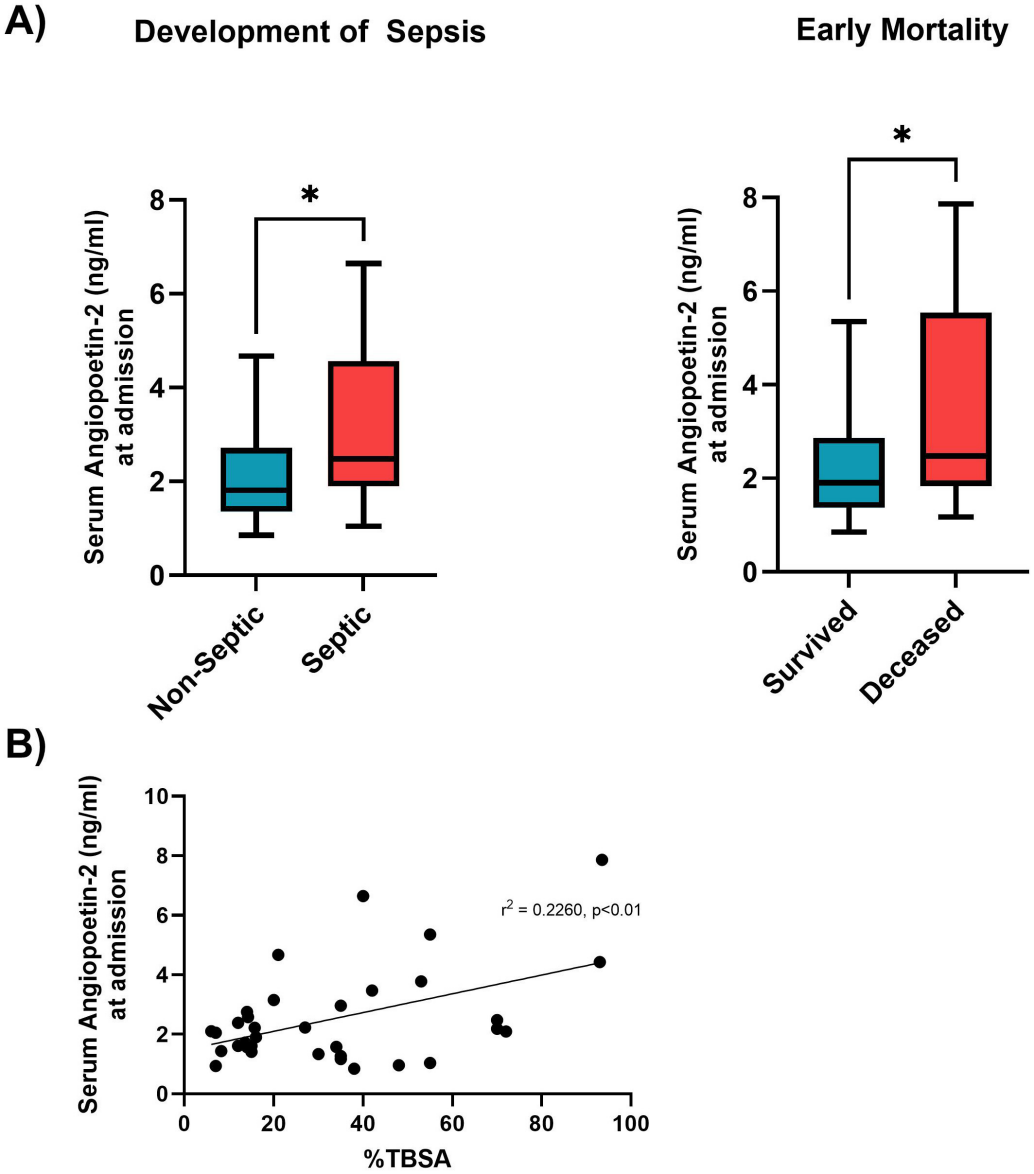# 813 Changes in Serum Angiopoietin-2 Levels Correlate with Sepsis and Mortality After Burn Injury

**DOI:** 10.1093/jbcr/iraf019.344

**Published:** 2025-04-01

**Authors:** Ryan Johnson, Kevin Galicia, Abigail Plum, Mary Grace Murray, Mashkoor Choudhry, John Kubasiak

**Affiliations:** Loyola University Chicago; Loyola University Chicago; Loyola University Chicago; Loyola University Chicago; Loyola University Chicago; Loyola University Chicago

## Abstract

**Introduction:**

Large burns induce a systemic inflammatory response, compromising vascular endothelium and hemodynamics, resulting in the degradation of the endothelial glycocalyx (EG), a luminal barrier regulating the passage of inflammatory molecules. EG breakdown releases Angiopoietin (Ang)-1 and Ang-2, essential mediators of vascular integrity in peripheral tissues and within the bone marrow by competing for the TIE2 receptor. Ang-2 acts to increase vascular permeability. potentially worsening capillary leak, tissue edema, and hypoxia. We propose that an early elevation of Ang-2 levels after burn injury leads to increased vascular permeability and tissue edema, which in turn causes hypoxia and elevates the risk of sepsis.

**Methods:**

Patients aged 18 and older who experienced burns involving ≥10%TBSA were enrolled in the study. Blood samples were collected at several time points for analysis using ELISA kits from various manufacturers and underwent flow cytometry for HSPC analysis. GraphPad (Prism) software utilized for statistical analysis.

**Results:**

There was a significant positive association between %TBSA and serum Ang-2 (sAng-2) levels (p< 0.05). Upon evaluation of the association between admission sAng-2 levels with the development of sepsis and early mortality, analysis showed significantly higher median sAng-2 levels in septic patients compared to non-septic patients (2.477 ng/ml vs. 1.812 ng/ml, p< 0.05). A similar trend was observed for early mortality, with higher median levels in those who did not survive compared to survivors (2.477 ng/ml vs. 1.904 ng/ml, p< 0.05) (Fig 1).

**Conclusions:**

Findings show a strong correlation between elevated sAng-2 levels shortly after burn injury and the risk of developing sepsis and early mortality in burn patients. Ongoing research aims to further investigate the underlying mechanisms. A timeline analysis comparing sAng-2 levels at admission with subsequent days is in progress, alongside additional analyses focused on survival outcomes in relation to angiopoietin-2 levels in patients with large burns.

**Applicability of Research to Practice:**

This research highlights the potential role of endothelial damage markers, such as sAng-2, in predicting the risk of sepsis and early mortality in burn patients. A panel of endothelial damage markers could be used to better assess vascular integrity and guide early interventions aimed at minimizing complications like sepsis, ultimately improving patient outcomes in burn care.

**Funding for the Study:**

This work is supported by the National Institute of General Medical Sciences (NIGMS: T32GM008750).